# Emerging Vistas for the Nutraceutical *Withania somnifera* in Inflammaging

**DOI:** 10.3390/ph17050597

**Published:** 2024-05-07

**Authors:** Vivek Basudkar, Gunjan Gujrati, Saiprasad Ajgaonkar, Manav Gandhi, Dilip Mehta, Sujit Nair

**Affiliations:** 1 PhytoVeda Pvt. Ltd., Mumbai 400 022, India; 2 Viridis Biopharma Pvt. Ltd., Mumbai 400 022, India; 3 College of Medicine, University of Illinois Chicago, Chicago, IL 60612, USA

**Keywords:** *Withania somnifera*, inflammaging, aging, healthy aging, inflammation, oxidative stress, DNA damage, immunomodulation, COVID-19, microbiome

## Abstract

Inflammaging, a coexistence of inflammation and aging, is a persistent, systemic, low-grade inflammation seen in the geriatric population. Various natural compounds have been greatly explored for their potential role in preventing and treating inflammaging. *Withania somnifera* has been used for thousands of years in traditional medicine as a nutraceutical for its numerous health benefits including regenerative and adaptogenic effects. Recent preclinical and clinical studies on the role of *Withania somnifera* and its active compounds in treating aging, inflammation, and oxidative stress have shown promise for its use in healthy aging. We discuss the chemistry of *Withania somnifera*, the etiology of inflammaging and the protective role(s) of *Withania somnifera* in inflammaging in key organ systems including brain, lung, kidney, and liver as well as the mechanistic underpinning of these effects. Furthermore, we elucidate the beneficial effects of *Withania somnifera* in oxidative stress/DNA damage, immunomodulation, COVID-19, and the microbiome. We also delineate a putative protein–protein interaction network of key biomarkers modulated by *Withania somnifera* in inflammaging. In addition, we review the safety/potential toxicity of *Withania somnifera* as well as global clinical trials on *Withania somnifera*. Taken together, this is a synthetic review on the beneficial effects of *Withania somnifera* in inflammaging and highlights the potential of *Withania somnifera* in improving the health-related quality of life (HRQoL) in the aging population worldwide.

## 1. Introduction

Aging is a time-associated natural and complex phenomenon leading to deterioration of physiological functions. A prominent aspect of aging involves control and gradual increase in proinflammatory status, termed as inflammaging [1]. Inflammaging is a chronic, systemic, low-grade inflammation resulting from high levels of inflammation that is primarily observed in elderly patients [2,3]. The term inflammaging was first given by Franceschi et al. [4] and involves numerous molecular and cellular mechanisms, such as cellular senescence, immunosenescence, mitochondrial, and ubiquitin-proteasome system dysfunction, activation of inflammasome, and DNA damage response [4,5]. Inflammaging can also contribute to the progression of various disease including cardiovascular disease [6], neurodegenerative disorders [7,8], diabetic nephropathy and diabetic retinopathy in diabetes [9,10,11,12], and cancer [13].

*Withania somnifera* or Ashwagandha, also referred to as winter cherry or Indian ginseng, which belongs to the Solanaceae family, is a well-known herb having numerous health benefits [14,15]. It is generally found in arid regions of South Asia, Central Asia and Africa; moreover, it is commonly used in Ayurveda (the Traditional Medicinal System of India) [16,17]. *Withania somnifera* has been used for thousands of years as a *‘Rasayana’* (*Rasayana* is a holistic therapy that helps by suppressing the aging process by developing positive physical and mental health, boosting the immune system and maintaining youthfulness [18]), along with providing numerous health benefits [19]. Furthermore, *Withania somnifera* is referred to as ‘*Avarada*’ in Ayurvedic literature, which indicates regeneration or youthfulness [20]. It is also included among the medicinal plants in the World Health Organization’s (WHO) monographs [21]. Phytoconstituents in *Withania somnifera* are present throughout the plant such as roots, leaves, and berries; however, the root section of *Withania somnifera* has the highest concentration of preferred bioactives. Withanolides and Withaferins (steroidal lactones), with saponins and alkaloids being a few of the active biological constituents present in *Withania somnifera* [22]. *Withania somnifera* has various properties, such as modulation of the immune system [23], antioxidant [24], antiinflammation [25], cardiorespiratory endurance [26], neuroprotection [27], male infertility treatment [28], anticancer activity [29], and decreases cortisol levels, stress, anxiety, and blood pressure in chronic stress.

The exact underlying mechanism of inflammaging is not completely known. However, there are various factors and causes that may lead to inflammaging. Studies have reported that inflammaging develops due to several factors, including oxidative stress, immunosenescence, cell senescence, hormonal changes, chronic infections, psychological stress, and metabolic changes. Earlier studies have discussed phytoconstituents and dietary supplements, such as curcumin from turmeric, ellagic acid from pomegranate, and epigallocatechin-3-gallate (EGCG) from green tea [30]; *Moringa oleifera* [31]; sandalwood oil [32]; sulforaphane [33]; and vitamin K2-7 [34,35], along with their numerous health benefits. Furthermore, the roles of various phytocompounds such as organosulfur compounds [36], EGCG [37], curcumin [38], flavonoids [39], soy isoflavone [40], and γ-tocopherol [41] in the modulation of gene expression for cancer have been reported. In addition, the role of phytocompounds when administered in combination with, for example, sulforaphane and EGCG for colon and prostate cancer [42,43] and curcumin and sulforaphane for cancer chemoprevention has been elucidated earlier [44]. In this review, we discuss the etiology of inflammaging and the protective role(s) of *Withania somnifera* in various organ systems. In addition, we elucidate the mechanistic basis for the health-beneficial effects of *Withania somnifera* using a systems pharmacology approach. Finally, we discuss clinical trials on *Withania somnifera* to collate a synthetic review on *Withania somnifera* in inflammaging.

## 2. Chemistry of *Withania somnifera*

**Figure 1:**
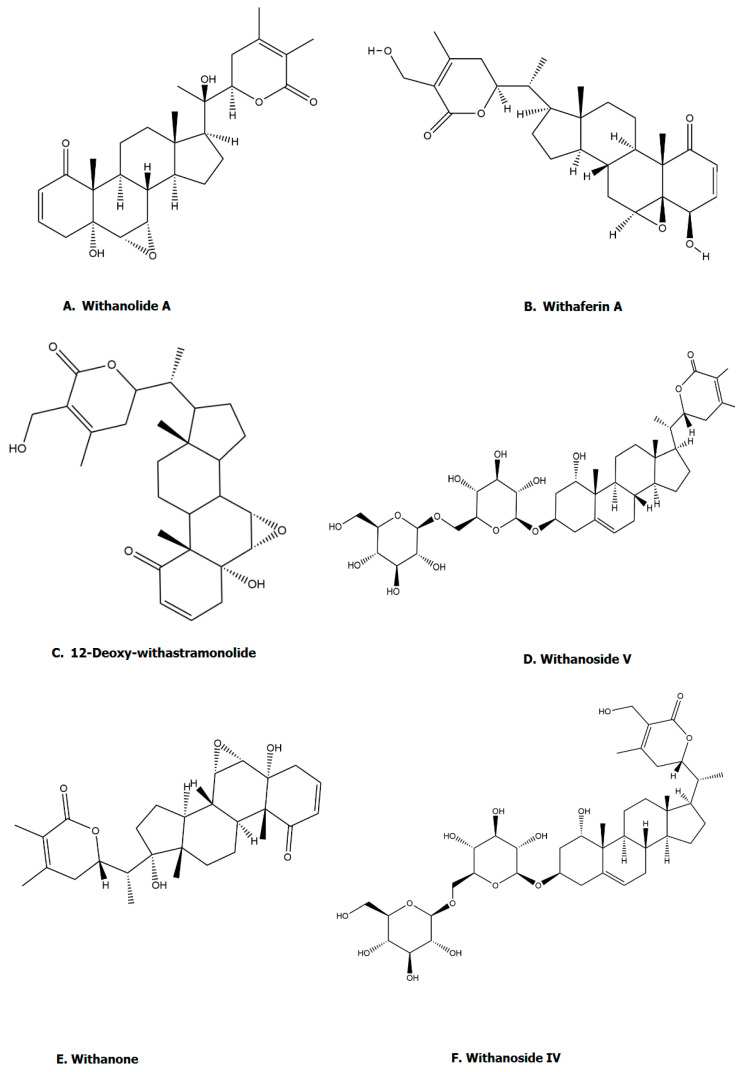

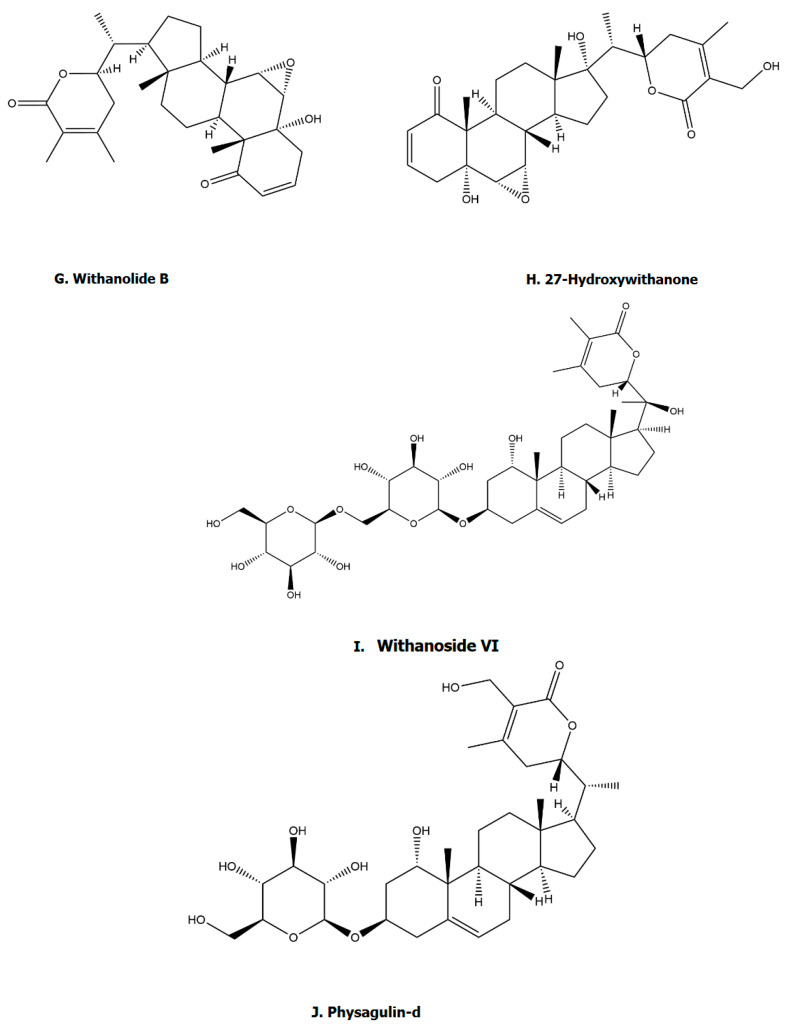
Active compounds of *Withania somnifera* (**A**) Withanolide A; (**B**) Withaferin; (**C**) 12-Deoxy-withastramonolide; (**D**) Withanoside V; (**E**) Withanone; (**F**) Withanoside IV; (**G**) Withanolide B; (**H**) 27-Hydroxywithanone; (**I**) Withanoside VI; (**J**) Physagulin-d. All the chemical structures were drawn using ChemDraw version 20.1.1.125.

*Withania somnifera* contains numerous active compounds, such as steroidal lactones (withanolides), alkaloids, and sitoindosides. Steroidal lactones, including withanolides and withanosides, are major phytoconstituents of *Withania somnifera*. *Withania somnifera* also contains sitoindosides, which are derivatives of withanolides (glucose at carbon 27 of withanolides) [45]. A study by Ghosal et al. reported that sitoindosides have CNS and immunomodulatory effects [46]. Akhoon et al. [20] have demonstrated improvement in life expectancy using Withanolide A in *Caenorhabditis elegans*. Various alkaloids are present in *Withania somnifera*, including anahygrine, cuseohygrine, anaferine, and isopellentierine. Choline, which is an important nutrient required in proper functioning of various physiological processes, is present in *Withania somnifera* [47,48]. *Withania somnifera* also contains tannins [49], which are polyphenolic compounds having various uses, such as antioxidant, antiinflammatory, anticancer, and neuroprotective. Moreover, *Withania somnifera* also contains carbohydrates, fatty acids, and amino acids that play a significant role in maintaining daily complex biochemical processes [50]. The chemical structures of Withanolide A, Withaferin, 12-Deoxy-withastramonolide, Withanoside V, Withanone, Withanoside IV, Withanolide B, 27-Hydroxywithanone, Withanoside VI, and Physagulin-d have been illustrated in Figure 1.

## 3. Etiology of Inflammaging

The precise etiology and underlying mechanisms involved in development and progression of inflammaging is currently unknown. Recent studies suggest a complex process that when understood can provide strategies for diagnosis and therapy of inflammaging associated with numerous factors, including cellular senescence, immunosenescence, oxidative stress, mitochondrial dysfunction, epigenetic changes, inflammasome activation, metabolic changes, microbiome alterations, DNA damage, microbiome alterations, genetic predispositions, lifestyle, and chronic diseases. Cellular senescence, a complex process driven by genetic, epigenetic, and environmental factors, affects many types of somatic cells, leading to progression of aging and chronic diseases. Senescent cells promotes proinflammatory and matrix-degenerating substances and further accumulation of senescent cells results in development of inflammaging [51]. Immunosenescence is a gradual process of deterioration of the immune system with increase in age. It contributes to the progression of inflammaging due to weakening of immune system that leads to various pathological conditions, such as dysregulation of immunomodulation, accumulation of senescent cells, failure in recognizing pathogens, and increase in autoimmune conditions [52].

## 4. *Withania somnifera* in Inflammaging

**Figure 2:**
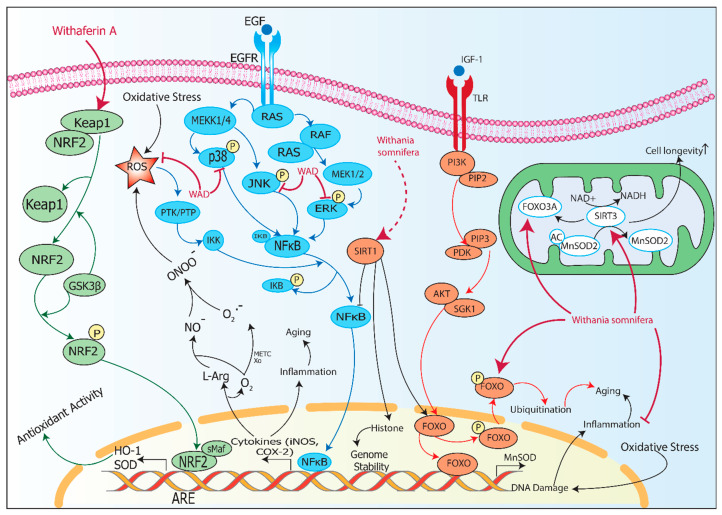
Molecular pathways for *Withania somnifera* in inflammaging. Withaferin A, an active compound of *Withania somnifera*, activates NRF2 by dissociating it from Keap1 which translocates to the nucleus and further binds to sMaf leading to transcription of antioxidant HO-1, which reduces oxidative stress. EGF binds to EGFR activating RAS/RAF pathway and phosphorylates ERK /p38/JNK; this further activates NF-κB, which transcribes cytokines that increase inflammation and aging. WAD inhibits p38, JNK, and ERK, while also suppressing ROS. SIRT1 can be activated by *Withania somnifera*, which inhibits NF-κB. IGF-1 binds to TLR and activates the PI3K/AKT pathway, which activates FOXO and transcribes MnSOD. Ubiquitination that leads to aging can be prevented by *Withania somnifera*. Also, *Withania somnifera* increases FOXO activity. Oxidative stress leads to DNA damage and inflammation, which can be reduced by *Withania somnifera*. *Withania somnifera* acts on FOXO3A and SIRT3, which helps in increasing cell longevity. Abbreviations: Keap1: Kelch like ECH associated protein 1; NRF2: Nuclear factor erythroid 2-related factor 2; GSK3β: Glycogen synthase kinase 3 beta; HO-1: Heme oxygenase-1; SOD: Superoxide dismutase; sMaf: Small musculoaponeurotic fibrosarcoma; ROS: Reactive oxygen species; ONOO^−^: Peroxynitrite; NO^−^: Nitric oxide; O_2_^.−^: Superoxide; L-Arg: Arginine; iNOS: Inducible nitric oxide synthase; COX-2: Cyclooxygenase-2; EGF: Epidermal growth factor; EGFR: Epidermal growth factor receptor; WAD: Withagenin A diglucoside; PTK/PTP: Protein tyrosine kinase/Protein tyrosine phosphatase; RAS: Rat sarcoma; RAF: Rapidly accelerated fibrosarcoma; JNK: c-Jun N-terminal kinase; ERK: Extracellular signal-regulated kinase; NF-κB: Nuclear factor-kappa B; IκB: Nuclear factor-kappa-B inhibitor; IGF-1: Insulin-like growth factor 1; TLR: Toll-like receptor; SIRT: Sirtuin; PI3K: Phosphatidylinositol 3-kinase; PIP2: Phosphatidylinositol 4,5-bisphosphate; PIP3: Phosphatidylinositol-3,4,5-trisphosphate; PDK: Pyruvate Dehydrogenase Kinase; AKT: Protein kinase; BSGK1: Serum/glucocorticoid regulated kinase 1; FOXO: Forkhead box O; NAD+: Oxidized nicotinamide adenine dinucleotide; NADH: Reduced nicotinamide adenine dinucleotide; MnSOD2: Manganese superoxide dismutase.

Inflammaging progression is associated with numerous factors, and hence, it is difficult to understand the complex process and to represent the role of *Withania somnifera* in mitigating it due to limited evidence. Here, we have discussed the role of *Withania somnifera* in inflammaging and the potential mechanisms involved in inflammaging (as illustrated in Figure 2). Grunz-Borgmann et al. [53] performed an experiment to study the role of *Withania somnifera* water extract in aging kidney with chronic inflammation using a rat proximal tubular cell line (NRK-52E). Monocyte chemotactic protein-1, also known as CCL2, belongs to the category of C-C chemokines and possesses properties such as chemoattractant for T lymphocytes, leukocytes, and natural killer cells (NK). CCL2 is overexpressed in patients with chronic kidney disease [54]. CCL5 belongs to the chemokine family that regulates T-cells, monocytes, eosinophils, and basophils in inflammation. Overexpression of CCL2 and CCL5 can play a key role in progression of fibrosis in chronic kidney disease. Grunz-Borgmann et al. demonstrated that *Withania somnifera* water extract suppressed the gene expression of CCL2 and CCL5 via TNF-α/LPS stimulation by reducing the activity of NF-kB.

### 4.1. Withania somnifera in Aging

The elasticity and strength of the human skin and body are maintained by collagen and elastin. In aged skin, release of MMP-1 (an enzyme that degrades collagen) and intracellular ROS production is induced by TNF-α. Lee et al. [55] isolated withagenin A diglucoside from methanolic extract of *Withania somnifera* and investigated its functions in TNF-α stimulated human dermal fibroblasts. It was observed that withagenin A diglucoside inhibits intracellular ROS production that leads to suppression in secretion of MMP-1 and collapsing collagen type 1. Withagenin A diglucoside demonstrated inhibition of phosphorylation of MAPK, Akt, c-JUN, NF-kB, and cyclooxygenase-2 (COX-2) expression. NF-kB is involved in cellular proliferation as well as inflammation via regulating gene expression [56]. Serine-threonine kinases, known as mitogen-activated protein kinases, or MAPKs, enhance intracellular signaling related to a variety of cellular processes, such as cell division, proliferation, survival, and transformation [57]. Akt stimulates mammalian cell survival and proliferation, helping in the development of tumors, and it has been demonstrated recently that Akt activity increases with cellular senescence and that primary cultivated human endothelial cells live longer when Akt is inhibited. Constitutive activation of Akt stimulates a p53/p21-dependent pathway that results in dysfunctional endothelial cells and senescence-like interruption of cell proliferation. Akt’s unique function in controlling cellular lifetime may be a factor in many human conditions [58]. The enzyme COX-2 in the prostaglandin synthesis pathway may have a role in elderly people by causing oxidative damage and various stress responses [59]. Also, it was observed that withagenin A diglucoside inhibited expression of pro-inflammatory cytokines IL-6 and IL-8, which demonstrated the potential use of withagenin A diglucoside in skin aging. Dysregulation of collagenous tissues occurs because of collagen cross-linking in aging and hyperglycemia conditions that specifically affects renal, cardiovascular, and retinal tissues, which contributes to severe illness or even death.

Babu et al. [60] demonstrated that *Withania somnifera* can significantly reduce glycation, AGE, and cross-linking compared to metformin. The ethanolic extract of *Withania somnifera* demonstrated greater efficacy than the root powder, suggesting that ethanolic extract contains more phytocompounds. Withanolide A promotes health, lowers age-related physiological changes, and increases lifespan. Akhoon et al. [20] showed that Withanolide A has various neuroprotective properties, including antiamyloidogenic actions, reduction of α-synuclein aggregation, and neuroprotection via regulation of neuronal mediators such as acetylcholine. Also, it was demonstrated that Withanolide A promotes stress tolerance and extends lifespan via the insulin/insulin-like growth factor signaling pathway. Numerous clinical ailments, including sarcopenia, are associated with aging. This is caused by a disparity in synthesis and breakdown of myofibrillary protein synthesis (particularly myosin heavy chain (MyHC) adult form), as well as a loss in muscle regenerative potential. Salvadori et al. [61] investigated the efficacy of WST formulation containing *Withania somnifera*, *Silybum marianum*, and *Trigonella foenum-graecum* against MyHC-II degradation for the protection of C2C12 myoblasts. It was observed that WST upregulates Akt (protein kinase B)-dependent protein synthesis as well as p38 MAPK (p38 mitogen-activated protein kinase)/myogenin-dependent myoblast differentiation. p38 MAPK found in mammals plays significant roles in muscle physiology [62]. WST also maintains trophism in both C2C12 and young myotubes, as well as restores sarcopenic myotube size, developmental myoblast fusion, and MyHC expression.

#### *Withania somnifera* in Healthy Aging

Forkhead box protein O3A (FOXO3A) and sirtuin 3 (SIRT3) are some of the important biomarkers that play key roles in longevity by regulating gene expression [63,64]. Pradhan et al. [65] performed an experiment to study the expression of FOXO3A and SIRT3 in healthy aging in humans (473 subjects) and HEK-293 cells. A significant decrease in FOXO3A and SIRT3 was observed with an increase in the age of the patients. Furthermore, in vitro studies performed in stress-induced HEK-293 cells treated with *Withania somnifera* showed upregulation of FOXO3A and SIRT3. Kuchewar et al. [66] demonstrated that oral administration of *Withania somnifera* (500 mg capsule twice a day) for 6 months showed significant decrease in malondialdehyde (MDA) and increase in superoxide dismutase (SOD) levels. Superoxide dismutase forms the front line of defense against reactive oxygen species-mediated injury; induction in SOD activity in human aging may be a compensatory response of the individual to increased oxidative stress [67]. Furthermore, it was also suggested that *Withania somnifera* can help in preventing premature aging. Nuclear factor erythroid 2-related factor 2 (Nrf2) provides cytoprotective action by modulating gene expression of numerous antioxidant enzymes via binding to the promotor region of antioxidant response element (ARE). However, it has been observed that with an increase in age, there is a decrease in activity of Nrf2 [68]. To activate the Nrf2 for promoting healthy aging, Hybertson et al. [69] used a combination of *Rosmarinus officinalis*, *Withania somnifera*, and *Sophora japonica* extract powder in the ratio 15:5:2 and treated human hepatocellular carcinoma HepG2 cells. The phytochemical combination of *Rosmarinus officinalis, Withania somnifera*, and *Sophora japonica* promoted the activation of NRF2, suggesting their potential role in treating aging.

Cabey et al. [70] studied the role of *Withania somnifera* water extract using an in vivo model (*Drosophila melanogaster*) and an in vitro model (HepG2 cells). It was reported that water extract of *Withania somnifera* showed significant increase in NRF2 levels in HepG2-ARE (HepG2 cells expressing antioxidant response element by the application of firefly luciferase gene) cells. Also, the sniffer *Drosophila* model of oxidative stress showed improvement in locomotion when treated with water extract of *Withania somnifera*. A similar study was carried out in *Drosophila melanogaster* by Holvoet et al. [71], which suggested that water and ethanol extract of *Withania somnifera* showed improvement in stress-induced behavioral changes. Also, it was observed that water extract of *Withania somnifera* was more potent than ethanol extract. This could be due to the higher concentration of withanolides in water extract. It has been observed that there is an increase in anxiety and depression with age.

A study performed by Singh et al. [72] in middle aged wistar female albino rats (11–12 months old) demonstrated that intermittent fasting-dietary restriction along with supplementation of *Withania somnifera* and *Tinospora cordifolia* extract showed decrease in expression of molecular stress chaperones Hsp70, which is responsible for inducing stress. It has also been reported that an increase in stress levels can result in upregulation of inflammatory cytokines. Molecular chaperones (Hsp70) play a key role in protein damage prevention throughout aging, and their expression is required for longevity. Chemical stimulation of HSP production could, thus, be an important method in the future formulation of antiaging drugs [73]. Study of cytokines levels in rats showed downregulation of IL-1β, IL-6, Iba1, and TNF-α [72]. IL-6, TNF-α, and C-reactive protein levels significantly increase in the elderly population and are associated with physical and cognitive performance and can lead to mortality [74]. TNF-α may affect glucose metabolism but aging and obesity prevent normal inhibition of TNF-α production, contributing to decreased insulin sensitivity in older men [75]. In an aging population, beta cell functioning and its renewal was influenced by IL-1β signaling molecule. Increased levels of IL-1β correspond to increased disabilities and mortality rates in geriatric populations [76].

Male reproductive diseases associated with male sexual potency and erectile dysfunction can be promoted due to psychological stress. *Withania somnifera* has the potential to improve male sexual health by promoting improvement in stress management, healthy aging, and body immune system. Yadav et al. [77] performed a study in sexually sluggish male rats previously exposed to psychological stressors by administering *Withania somnifera* powder extract for 30 days. It was observed that *Withania somnifera* promoted the expression of cGMP, acetylcholine, nitric oxide, eNOS, and nNOS, which are associated with penile erectile facilitation. The incidence of penile erectile dysfunction increases with aging due to reduced nitric oxide synthesis, which is caused by decreased NOS expression and reduced endothelial NOS and neuronal NOS activity. This dysregulation leads to oxidative stress which results in an aged penis [78,79]. Also, there was an increase in serum levels of luteinizing hormone, follicle-stimulating hormone, and testosterone. The study concluded that *Withania somnifera* can be used as a potential phytochemical to improve sexual health in stressed, sexually sluggish male rats. Similarly, Gupta et al. [80] performed an experiment in 180 infertile male patients to study the role of *Withania somnifera*. Patients were administered with *Withania somnifera* powder extract (5 g/d for 3 months), which showed improvement in concentration of phenylalanine, histidine, glycerylphosphorylcholine, citrate, alanine, and lactate in seminal plasma.

Kukkemane et al. [81] performed an experiment to demonstrate the role of *Withania somnifera* against age-related suprachiasmatic nucleus changes. The experiment was performed in male wistar rats administered with 100 mg/kg of *Withania somnifera* leaf extract. Studies suggested that leaf extract of *Withania somnifera* can modulate core clock genes expression via SIRT1. SIRT1 is a circadian clock regulator, resulting in the restoration of rhythm/levels in the expression of clock genes. Such changes could restore the rhythmic production of Nrf2, a clock-controlled gene and master transcription factor that regulates antioxidant enzymes. Furthermore, naturally occurring antioxidant enzymes, as well as the possible antioxidants of *Withania somnifera* leaf extract, may neutralize reactive oxygen species. These results suggest that *Withania somnifera* leaf extract can alleviate age-related circadian disruption, perhaps leading to healthy aging and longevity.

### 4.2. Withania somnifera in Inflammation

Inflammation is a body’s immune system response to external stimuli by pathogens or injury that involves a complex biological process. There are various factors associated with inflammation, such as infections, chronic stress, environmental toxins, aging, genetics, hormones, autoimmune disorders, metabolic condition, etc. However, recent studies have suggested that various complex phenomena, such as accumulation of senescent cells, oxidative stress, dysfunction of mitochondria, hormonal changes, nutrition, and genetics due to aging, contribute to progression of inflammation. Sikandan et al. [82] investigated the antiinflammatory role of *Withania somnifera* water extract in human keratinocyte HaCaT cells and male C67BL/6J mice. It was found that *Withania somnifera* significantly suppressed inflammatory cytokines TNF-α, IL-1β, IL-6, IL-8, and IL-12 and overexpressed TGF-β1. Moreover, it was observed that *Withania somnifera* also inhibited NF-ΚB p65, c-Jun N-terminal kinase, and phosphorylation of p38. Also, suppression of TNF-α and upregulation of TGF- β1 were demonstrated in in vivo studies. IL-8 (a potent chemoattractant) is involved in recruiting immune cells to inflammation site [83]. Hence, increased IL-8 levels have been detected in several age-related complications mainly associated with inflammation [84]. Neuroinflammation promoted by prolonged activation of IL-12 and tumor growth factor-β1 are associated with neurodegenerative disorders such as Alzheimer’s disease and mild cognitive impairment [85,86].

#### 4.2.1. *Withania somnifera* in Neuroinflammation

Neuroinflammation is associated with aging that can lead to various neurological disorders, including Alzheimer’s disease [87]. A study was performed by Gupta et al. [88] in wistar strain male albino rats to understand the underlying role of *Withania somnifera* leaf water extract in treating neuroinflammation. It was demonstrated that *Withania somnifera* leaf water extract suppressed cytokines associated with inflammation such as TNF-α, IL-1β, IL-6, reactive nitrogen species, and reactive oxygen species in the brain. Furthermore, it was observed that *Withania somnifera* leaf water extract suppressed NF-kB, p38, and JNK pathways. Similarly, another study performed by Gupta et al. [89] in primary microglial culture and mouse microglial BV-2 cell line showed suppression of IL-6, IL-1β, TNF-α, reactive nitrogen species, and a reactive oxygen species by downregulating NF-kB and AP1.

Withaferin A, an active constituent of *Withania somnifera* showed inhibition of NF-kB and REL A transcription factors. Also, overexpression of IKBKB and IKBKG (subunits of NF-kB) along with downregulation of JUN and STAT genes were observed in co-cultured cells of SH-SY5Y transfected with amyloid precursor protein plasmid (SH-APP) and immortalized CHME5 microglia cells [90]. Zhu et al. [91] performed an experiment in adult male C57BL/6 mice to investigate the role of withanolide A, a bioactive compound in *Withania somnifera*, in pilocarpine-induced status epilepticus. It was observed that withanolide A can suppress IL-1β and TNF in the hippocampus region of the mice. Furthermore, it was concluded withanolide A can provide neuroprotective effects by inhibiting neuroinflammation.

Microtubule associated protein 2 (MAP2) and growth associated protein 43 (GAP43) gene expression helps in maintenance of neuronal functions. An experiment carried out by Gupta et al. [92] in wistar strain male albino rats observed significant downregulation of MAP2 and GAP43 in LPS-mediated inflammation. Also, it was observed that Ashwagandha leaf water extract may protect neurons from neuroinflammation-associated degeneration by controlling the expression of microtubule associated protein 2 and growth associated protein 43. Amphetamine-related drug (+/−)-3,4-methylendioxymethamphetamine (MDMA) is used in treating neurological disorders. However, prolonged use of MDMA can lead to various disorders, including neuroinflammation. An experiment was performed in adult male C57BL/6J mice by Costa et al. [93], to determine potential benefits of *Withania somnifera* extract in suppressing MDMA-induced toxicity. It was concluded that animals treated with MDMA along with *Withania somnifera* extract prevented dopaminergic damage. The dopaminergic system in the brain can alter significantly with age, which may have an impact on cognitive performance. Dopamine levels in the human striatum can drop by up to 50% as people age, making the dopamine system particularly susceptible to the effects of aging [94].

#### 4.2.2. *Withania somnifera* in Lung Inflammation

Lung aging occurs due to various factors, including improper intercellular communication, ECM dysregulation, genomic instability, epigenetic modification, telomere attrition, proteostasis loss, mitochondrial dysfunction, cellular senescence, stem cell exhaustion, and deregulated nutrient-sensing. These factors can contribute in the progression of lung inflammation [95]. Kaur et al. [96] demonstrated an increase in the level of IL-10 and a decrease in TNF-α and NF-kB in lung homogenate of monocrotaline-induced Sprague Dawley rats treated with *Withania somnifera* root powder. Decreasing levels of IL-10 is closely associated to aging-related inflammation, because it facilitates reducing the inflammation of the skeletal muscles [97].

#### 4.2.3. *Withania somnifera* in Kidney Inflammation

Inflammation and premature aging are a few of the major contributing factors in the progression of chronic kidney disease [98]. Chen et al. [99] treated male C57BL/6J mice model of unilateral ureteral obstruction (UUO) with Withaferin A. Levels of inflammatory markers p-NF-kB-p65, IL-1β, and COX-2 were suppressed in UUO animals treated with Withaferin A for 14 days.

#### 4.2.4. *Withania somnifera* in Liver Inflammation

The aging process often contributes to modulation in mitochondrial function and nutrient sensing pathways that can lead to inflammation and cellular senescence [100]. Xia et al. [101] investigated the role of Withaferin A in fulminant hepatitis (FH) in liver. In this experiment, C57BL/6J mice were administered with D-galacosamine (GaLN)/lipopolysaccharide (LPS) to induce FH. Withaferin A inhibited hepatic NLRP3 and upregulated the expression of NRF2. Furthermore, it also suppressed the release of hepatic IL-1β, IL-6, and TNF-α. These results suggested that Withaferin A can be used in the treatment of fulminant hepatitis as an immunoregulator. Aging-related NLRP3 inflammasome activation and hyperactivation have been directly linked to inflammatory reactions and cellular damage. Indeed, research has indicated that the activation of the NLRP3 inflammasome leads to intracellular inflammation, which is implicated in the process of aging [102]. Liver X receptors (LXR) are cholesterol-sensing nuclear receptors that regulate metabolism and transportation of lipids. Furthermore, they suppress inflammation by transrepression mechanism [103]. Also, it has been reported that LXR-α negatively modulates NF-kB signaling pathway. Shiragannavar et al. [104] studied the mechanistic role of Withaferin A in HepG2, Hep3B, and Huh-7 hepatocellular carcinoma cells in modulating expression of various genes. It was demonstrated that Withaferin A activated LXR-α that led to suppression of NF-kB, eventually leading to the attenuation of inflammation. Sirtuin 3 (SIRT3) is a deacetylase enzyme that plays a critical role in developing numerous inflammatory diseases, such as liver fibrosis. Gu et al. [105] revealed that Withaferin A activated SIRT3, which suppressed liver fibrosis via upregulating the activity of antioxidant enzymes such as catalase (CAT), superoxide dismutase, and glutathione peroxidase (GPx). Indeed, GPx plays a critical role in the reduction of hydrogen peroxide that contributes to aging by activating NF-κB-related inflammatory signaling and tissue damage [106]. Devkar et al. [107] investigated the hepatoprotective effect of *Withania somnifera* in acetaminophen-overdosed adult male albino wistar rats. Animals were treated with 750 mg/kg of acetaminophen for 14 days preceded by administration of withanolide rich fraction isolated from *Withania somnifera* root methanolic extract. Results indicated significant suppression of inflammatory markers such as IL-1β, TNF-α, COX-2, and iNOS in animals treated with withanolide rich fraction. Hence, it was concluded that withanolide rich fraction can have the potential to exert hepatoprotective action by suppressing oxidative stress and inflammation.

Although there is limited clinical evidence on the role(s) of *Withania somnifera* in neuroinflammation, lung inflammation, kidney inflammation, and liver inflammation, the above-mentioned preclinical studies exploring its effects on these inflammatory conditions offer valuable insights. These studies lay the groundwork for conducting better-informed clinical research to investigate the potential therapeutic benefits of *Withania somnifera.*

## 5. Inflammaging and Oxidative Stress: Protective Role of *Withania somnifera*

Oxidative stress is a state due to imbalance in reactive oxygen species (ROS) and plays a critical role in progression of inflammaging. ROS being highly reactive in nature contributes in inflammaging by damaging cellular components such as DNA, protein, and lipids, causing endoplasmic reticulum stress, and activating inflammatory pathways and senescence-associated secretory phenotype (SASP). Bakar et al. [108] performed an experiment to determine the role of Withaferin A in male C57BL/6J obese mice associated with oxidative stress and inflammation. It was demonstrated that Withaferin A suppresses oxidative stress by maintaining the levels of superoxide dismiutase, glutathione peroxidase, glutathione (GSH), thiobarbituric acid reactive species (TBARS), and catalase. Catalase is essential for maintaining cellular homeostasis because of its capacity to break down hydrogen peroxide, the absence or dysregulation of which is associated with several age-related complications [109]. The antioxidant glutathione, which helps with drug and xenobiotic detoxification strengthens anomalies of the liver, diminishes the complications of diabetes, and protects against viral infections [110]. The role of Withaferin A in cerulein-induced acute pancreatitis was studied by Tiruveedi et al. [111] in adult male Swiss mice. Withaferin A suppressed myeloperodixase (MPO), a peroxidase enzyme and nitrotyrosine and upregulated Nrf2 in acute pancreatitis mice indicating its potential role as an antioxidant. MPO levels are positively correlated with type 2 diabetes, a major risk factor for cardiovascular illnesses and mortality, whereas reduced MPO levels are associated with improved survival in the elderly [112]. To determine the role and underlying mechanism of *Withania somnifera* in rheumatoid arthritis, Khan et al. [113] performed a study in male Wistar albino rats. This rodent model with rheumatoid arthritis was treated with *Withania somnifera* water extract and evaluated for various parameters including the measurement of inflammatory cytokines and antioxidant markers. Furthermore, antioxidant superoxide dismutase activity was increased whereas catalase and glutathione peroxidase were suppressed indicating the antioxidant property of *Withania somnifera*.

## 6. Inflammaging and DNA Damage: Protective Role of *Withania somnifera*

Human cells, during their lifespan, undergo DNA damage, which is defined as changes in molecular structure of genomic DNA [114]. DNA damage occurs due to various endogenous (DNA replication errors, spontaneous base deamination, abasic sites, oxidative DNA damage, DNA methylation) and exogenous factors (ionizing radiation, ultraviolet radiation, chemical agents such as alkylating agents, toxins, environmental stresses, DNA damage response) [115]. Numerous theories of DNA damage and DNA repair have suggested that DNA damage can lead to the development of aging and contribute to inflammaging [116,117]. DNA damage response is a complex phenomenon that leads to coordination of cell cycle arrest and activation of repair mechanisms to prevent replication of damaged DNA. Persistent DNA damage response can lead to cell senescence and contribute to chronic inflammation. Senescence-associated secretory phenotype is activated due to chronic DNA damage response and senescent cells lead to release of various proinflammatory cytokines and chemokines, which can contribute to the progression of inflammaging. Also, the activation of immune response due to cell senescence with damaged DNA can further contribute to the progression of inflammaging. Furthermore, chronic inflammation can increase DNA damage by generating ROS and reactive nitrogen species (RNS). The impaired cell function due to accumulation of damaged DNA will further increase proinflammatory cytokines production and continuously maintain chronic inflammatory state. Oxidative stress is one of the major causes of DNA damage and *Withania somnifera* has been used as an antioxidant agent in treatment of numerous diseases. Hence, *Withania somnifera* can be used as a potential candidate for suppression of DNA damage that may help in prevention of inflammaging.

## 7. Inflammaging and Immunomodulation: Protective Role of *Withania somnifera*

Immunomodulation is a process that involves the regulation of immune system, which can play a critical role in mitigating inflammaging. Immunomodulation is performed by maintaining the level of inflammatory response, regulation of immune cells (macrophages, B cells, and T cells), cytokine regulation, and suppression of immunosenescence effect. *Withania somnifera* plays an important role in immunomodulation, with clinical evidence suggesting the role of *Withania somnifera* for the same [118,119]. Also, *Withania somnifera* helps in increasing the levels of white blood cells that helps in the development of human immune defense. Furthermore, it is used in treating arthritis [120]. *Withania somnifera* suppresses delayed type hypersensitivity and increases the macrophage phagocytic activity. Also, *Withania somnifera* increases the nitric oxide signaling in macrophages by stimulating nitric oxide synthase enzyme activity [121]. Tharakan et al. [122] reported the immunomodulatory effect of *Withania somnifera* in a randomized placebo-controlled double-blinded with an open-label extension study for 30 days in 24 adult male and female healthy volunteers administered with 60 mg of *Withania somnifera* extract. It was reported that *Withania somnifera* improved immunity by demonstrating significant increase in IgA, IgM, IgG, IgG2, IgG3, IgG4, IFN-γ, IL-4, CD45+, CD3+, CD4+, CD8+, CD19+, and NK cells. Immunoglobulins, a group of glycoproteins, play a vital role in the immune system. However, the level of IgM (which plays a key role in immune response to pathogens) significantly decreases with age while the level of IgA and IgG increases [123]. IFN-γ and IL-4 are key components of the immune response. IFN-γ promotes cellular immunity against intracellular infections, whereas IL-4 promotes humoral immunity and allergy responses. The balance of these cytokines is critical for immunological homeostasis and proper response to varied immune stressors. IFN-γ and IL-4 dysregulation is linked to a range of immunological disorders, including infections, autoimmune, and allergy problems [124]. Changes in CD45 expression and activity have been observed with aging. CD45 expression increases in healthy aged individuals compared to younger individuals [125]. Priyanka et al. [126] reported suppression of IL-6 suggesting immuno-stimulant properties of *Withania somnifera* in stress-induced equines in a study carried out for 21 days. A study was performed by Mikolai et al. [127] in which five volunteers were administered with 6 mL *Withania somnifera* root extract for 96 h. Expression of CD4 on CD3+ T cells and activation of CD56+ NK cells after 96 h in peripheral blood samples was observed. The protective effect of *Withania somnifera* was investigated in albino mice by Ziauddin et al. [128], who showed that *Withania somnifera* can be used an immunomodulatory agent to protect against harmful effects of myelosuppressive drugs.

## 8. Inflammaging and Microbiome: Protective Role of *Withania somnifera*

The microbiome plays an important role in human health. The microbiome and host human go through numerous physiological changes in aging. These changes can contribute to regulating the immune system, thereby influencing inflammation. Variations in the composition, function, phenotype, diversity, and metabolic output of the microbiome are significant age-dependent alterations that can significantly affect the homeostasis and immune balance. These shifts in the microbiome can contribute to inflammaging, leading to an increased inflammatory responses and excessive adverse effects. In 2015, Clark et al. [129] investigated the impact of microbiota on *Drosophila*, focusing on heightened intestinal permeability, inflammation, and mortality. Another study in 2016 by Conley et al. [130] explored microbial dysbiosis associated with aging in mice, leading to increased intestinal permeability and subsequent systemic inflammation.

Toll-like receptor protein (TLR4), which is present on the surface of various cells, including the gut, plays a critical role in the innate immune system. Danger-associated molecular patterns (DAMPs) and pathogen-associated molecular patterns (PAMPs) activate TLR4. Lipopolysaccharides (LPS) present on the surface of cell wall of bacteria activate TRL4. In the case of aged patients, TLR4 gets continuously activated due to the intake of bacterial products. Purushotham et al. reported that active phytocompounds of *Withania somnifera* inhibited TLR-4-activated innate immunity signaling pathways, including MAPK and NF-κB [131].

## 9. Inflammaging and COVID-19: The Protective Role of *Withania somnifera*

The severe acute respiratory syndrome coronavirus (COVID-19) pandemic that led to the disruption of normal life severely affected the elderly population. According to the Centers for Disease Control and Prevention, more than 80% of deaths caused by COVID-19 were above the age of 60 years [132]. Inflammaging contributes to various age-associated disorders that increase the risk of COVID-19 disease [133]. These disorders include diabetes, neuropathic degeneration, and rheumatoid arthritis. Furthermore, COVID-19 worsens when it co-exists with these diseases and can also become fatal [133,134,135,136]. Inflammaging can also contribute to the progression of cytokine storm, which is a complex of acute systemic inflammatory symptoms, elevated cytokine levels, and secondary organ dysfunction [137]. SARS-CoV-2 can be triggered by the presence of pro-inflammatory environments in patients suffering from inflammaging. NF-κB is a crucial regulator of inflammation that can contribute to exaggerating the inflammatory response in older people who have inflammation when they contract the virus. Hence, targeting NF-κB signaling may be used as a potential therapeutic approach for COVID-19. Downregulation of NF-κB can help in reducing the severity of cytokine storm. Numerous preclinical studies suggest the role of *Withania somnifera* in downregulation of NF-κB [53,82,138,139]. Also, the level of pro-inflammatory cytokines, including TNF-α and IL-6, that are observed in cytokine storm can be regulated by *Withania somnifera* suggesting the potential role of *Withania somnifera* in managing disease progression associated with COVID-19. Interferon (IFN) proteins play a critical role in immune response. They exist in different forms, i.e., IFN-α, IFN-β, IFN-γ, and IFN-λ, depending on the bodily innate immune response to viral infections. There is dysregulation of IFN response in elderly people including patients suffering from inflammaging that can lead to severe harmful conditions in patients with COVID-19. A study performed by Davis et al. [140] demonstrated enhanced levels of IFN-γ in mice treated with *Withania somnifera* root extract, suggesting their potential role.

**Figure 3:**
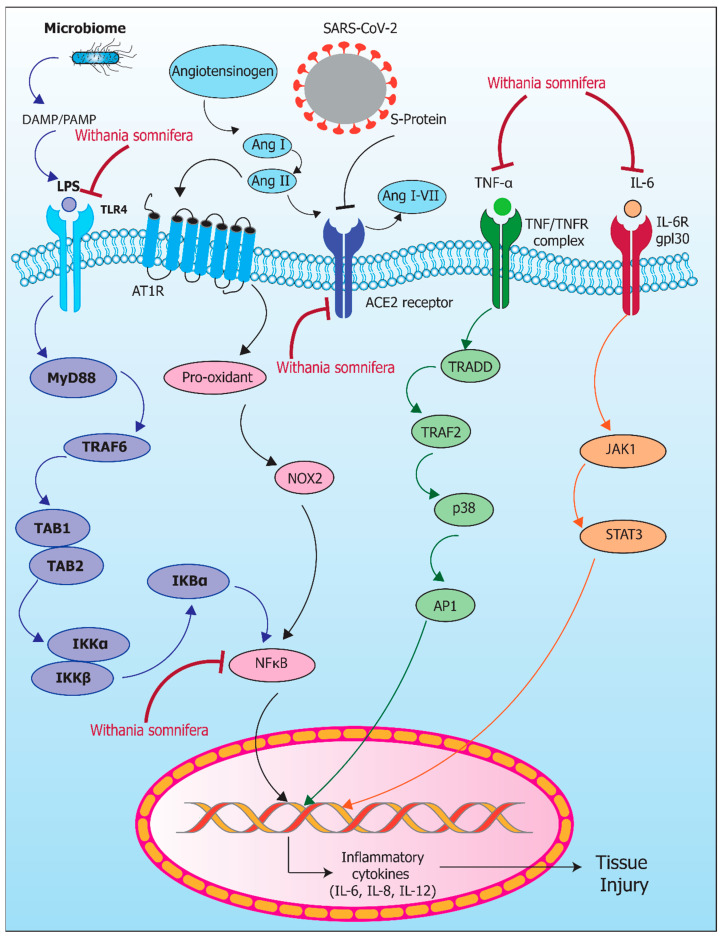
Molecular pathways for *Withania somnifera* in inflammaging modulated by COVID-19 and the microbiome. Microbiome releases LPS that activate TLR4 that initiates the activation of NF-κB pathway via MyD88 and IKBα. *Withania somnifera* inhibits TLR4 receptor as well as NF-κB. Angiotensinogen converts Ang I to Ang II, while Ang II activates the AT1R receptor; this also leads to activation of NF-κB via pro-oxidants and NOX2. Ang II activates Ang I–VII via the ACE receptor. S-protein of SARS-CoV-2 binds to ACE2 receptor to enter the cell; *Withania somnifera* inhibits the ACE2 receptor. TNF-α binds to TNFR receptor and activates the TRADD/TRAF pathway, which upregulates AP1 via p38 and transcribes inflammatory cytokines. IL-6 binds to IL-6R and activates the JAK1/STAT3 pathway, which leads to tissue injury by release of inflammatory cytokines. *Withania somnifera* inhibits TNF-α and IL-6, which help in the suppression of tissue injury. SARS-CoV-2: Severe Acute Respiratory Syndrome Coronavirus 2; DAMP: Damage-Associated Molecular Pattern; PAMP: Pathogen-Associated Molecular Pattern; Ang: Angiotensin; LPS: Lipopolysaccharide; S-Protein: Spike Protein; TLR4: Toll-like Receptor 4; Ang I: Angiotensin I; Ang II: Angiotensin II; Ang I-VII: Angiotensin I-VII; AT1R: Angiotensin II Receptor Type 1; ACE2 receptor: Angiotensin-Converting Enzyme 2 Receptor; IL-6: Interleukin-6; TNF-α: Tumor Necrosis Factor Alpha; IL-6R gp130: Interleukin-6 Receptor Glycoprotein 130; TNF/TNFR complex: Tumor Necrosis Factor/Tumor Necrosis Factor Receptor Complex; MyD88: Myeloid Differentiation Primary Response 88; Pro-oxidant: Substances promoting oxidative stress; TRAF2: Tumor Necrosis Factor Receptor Associated Factor 2; TRADD: Tumor Necrosis Factor Receptor Type 1-Associated DEATH Domain Protein; TRAF6: Tumor Necrosis Factor Receptor Associated Factor 6; p38: mitogen-activated protein kinases; NOX2: NADPH Oxidase 2; JAK1: Janus Kinase 1; TAB1: TGF-beta-Activated Kinase 1-Binding Protein 1; TAB2: TGF-beta-Activated Kinase 1-Binding Protein 2; IKBα: Inhibitor of Nuclear Factor Kappa-B Kinase Subunit Alpha; IKBβ: Inhibitor of Nuclear Factor Kappa-B Kinase Subunit Beta; NF-κB: Nuclear Factor Kappa-Light-Chain-Enhancer of Activated B Cells; STAT3: Signal Transducer and Activator of Transcription 3; AP1: Activator Protein 1; ROS: Reactive Oxygen Species; IL-6: Inflammatory cytokine-6; IL-8: Inflammatory cytokine-8; IL-12: Inflammatory cytokine-12.

Angiotensin converting enzyme (ACE2) plays a critical role in the regulation of blood pressure, sodium, and fluid balance. The spike protein of SARS-CoV-2 binds to the ACE2 receptor and facilitates the progression of COVID-19. In the renin-angiotensin-aldosterone system, the ACE2 receptor plays an important role in the regulation of inflammation. Angiotensin II is converted to angiotensin 1-7 by ACE2, which reduces the proinflammatory effects including IL-6, TNF-α, and IL-8 by inhibiting P38 mitogen-activated protein kinase (MAPK)-NF-kB pathway. ACE2 has antiinflammatory properties which protects the lungs from injury during acute respiratory distress syndrome (ARDS). Severe COVID-19 cytokine release during SARS-CoV-2 infection is partially triggered by increased angiotensin II, possibly as a result of TNF-α overproduction [141]. Indeed, the underlying mechanism is not completely understood. There are studies which suggest that *Withania somnifera* can modulate ACE2 that can be used as a potential approach for treating COVID-19; however, more research is required to prove the role of *Withania somnifera* in modulation of ACE2 in COVID-19 [142]. In Figure 3, we have depicted the molecular pathways for *Withania somnifera* in inflammaging that are modulated by COVID-19 and the microbiome.

The present discussion that *Withania somnifera* may potentially play a protective role in mitigating the detrimental effects of inflammaging during COVID-19 is, indeed, speculative given the absence of data at the time of writing. Further preclinical and clinical studies are necessary to validate this proposition.

**Figure 4:**
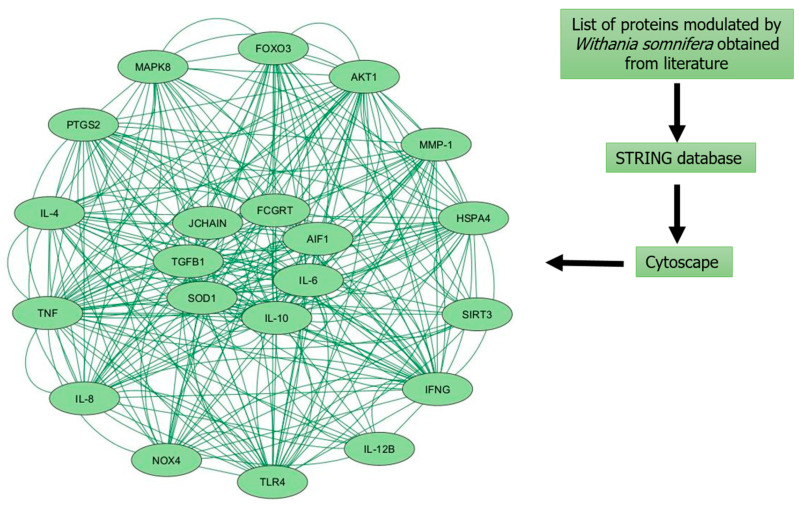
*Withania somnifera*-modulated putative protein–protein interaction network in inflammaging. Protein–protein interaction (PPI) network comprising upregulated and downregulated proteins modulated by *Withania somnifera* in inflammaging. The PPI with 21 nodes and 262 edges was constructed using STRING database [143] and Cytoscape version 3.10.1 [144]. FOXO3: Forkhead box O3; MAPK8: Mitogen-activated protein kinase 8; AKT1: RAC-alpha serine/threonine-protein kinase; PTGS2: Prostaglandin-endoperoxide synthase 2; MMP-1: Matrix metalloproteinase-1; IL-4: Interleukin-4; TNF: Tumor necrosis factor; IL-8: Interleukin-8; JCHAIN: Joining chain of multimeric IgA and IgM; HSPA4: Heat shock 70 kDa protein 4; TGFβ1: Transforming growth factor beta-1; SOD1: Superoxide dismutase; FCGRT: Fc fragment of IgG receptor and transporter; AIF1: Allograft inflammatory factor 1; IL-6: Interleukin-6; IL-10: Interleukin-10; SIRT3: Sirtuin-3; IFN_γ_: Interferon-gamma; NOX4: NADPH oxidase 4; IL-12β: Interleukin-12 subunit beta; TLR4: Toll-like receptor 4.

In addition, we have constructed a protein–protein interaction network, illustrated in Figure 4, for inflammaging modulated by *Withania somnifera*. A protein–protein interaction network was constructed using *Withania somnifera-*modulated proteins obtained from the literature, which was filtered using the STRING database and illustrated using Cytoscape 3.10.1. This biological network, which is focused on *Withania somnifera-*modulated proteins linked to inflammaging, unveils potential targets and key *Withania somnifera-*regulated pathways, offering valuable insights into therapeutic avenues for age-related inflammation. Furthermore, we have summarized various preclinical studies implicating health-beneficial effects of *Withania somnifera* in inflammation and aging in Table 1 for the benefit of the reader.

## 10. Safety and Toxicity of *Withania somnifera*

Recent investigations suggest the efficacy of *Withania somnifera* in numerous diseases. Clinical trial studies have been performed to determine the safety and efficacy of *Withania somnifera*. A pilot study was performed by Sharma et al. [161] with the objective to investigate the safety and efficacy of *Withania somnifera* in 25 hypothyroid patients. Studies demonstrated no significant changes between the placebo and *Withania somnifera*-treated groups indicating the safety of *Withania somnifera*. Likewise, Verma et al. [162] performed a study to investigate safety and tolerability in 80 healthy volunteers. *Withania somnifera* root extracts were administered orally 300 mg twice daily for 8 weeks. Studies reported no significant change in body weight, body temperature, body mass index, respiratory rate, systolic and diastolic blood pressure, hematological and biochemical parameters, and thyroid hormone, suggesting that *Withania somnifera* root extract is well-tolerated in healthy male and female volunteers for 8 weeks. Langade et al. [163] demonstrated safety of *Withania somnifera* in healthy volunteers and insomnia patients treated with *Withania somnifera* root extract capsules 300 mg twice daily for 8 weeks.

## 11. Clinical Studies on *Withania somnifera*

There are various global clinical trials carried out on *Withania somnifera*, including aging and safety studies. The opportunities and challenges in resource-limited clinical trials have been discussed elsewhere [164]. Herein, in Table 2, we have summarized the various clinical trials adapted from www.clinicaltrials.gov (accessed on 26 December 2023) [165], Australia New Zealand Clinical Trial Registry www.anzctr.org.au/ (accessed on 26 December 2023) [166], and International Clinical Trial Registry Platform www.who.int/clinical-trials-registry-platform (accessed on 26 December 2023) [167] for the benefit of the reader. However, there are limited results available from clinical studies, and almost none of these trial results have been subjected to peer review, thus underscoring their uncertain status.

## 12. Conclusions and Future Perspectives

*Withania somnifera*, a traditional Ayurvedic medicine, has numerous health benefits, including its abilities as an antioxidant and antiinflammatory agent. There are numerous preclinical and clinical studies attempting to shed light on *Withania somnifera*’s potential significance in the treatment of inflammation, neurological disorders, aging, healthy aging, and fertility problems. However, conclusive evidence regarding its therapeutic potential is yet to be established in clinical studies. The aging-associated phenomenon known as inflammaging, prevalent among the elderly, results from multifaceted factors. *Withania somnifera* has the potential to treat inflammaging, as there is considerable evidence that demonstrates its efficacy. *Withania somnifera* possesses antioxidant properties that help retard the progression of inflammaging. Furthermore, the antiinflammatory property of *Withania somnifera* contributes to modulating the chronic low-grade inflammation characteristic of this aging-related disorder. Additionally, antiaging effects, involvement in DNA damage repair mechanisms, and potential effects on the microbiome are factors that suggest a key role for *Withania somnifera* in inflammaging. However, there is limited preclinical and clinical evidence to firmly support this hypothesis. Hence, more studies are required to be carried out to understand the therapeutic efficacy and mechanism of *Withania somnifera* in treating inflammaging. Herein, we have discussed the etiology of inflammaging and the chemistry of *Withania somnifera*. Furthermore, we have illustrated the biological pathways involved in inflammaging that are regulated by *Withania somnifera* and its active compounds which may help in providing insights into the potential role(s) of *Withania somnifera* in inflammaging. Also, a protein–protein interaction network was generated in order to have a systems pharmacology perspective of key proteins modulated by *Withania somnifera* and to help identify key biomarkers as well as provide potential targets for treating inflammaging. This review underscores the potential health-beneficial effects of *Withania somnifera* in inflammaging and provides hope to improve the health-related quality of life (HRQoL) in aging.

## Figures and Tables

**Table 1:** Preclinical studies for *Withania somnifera* in inflammation and aging.

Sr No.	*Withania somnifera* Source	Preclinical Model	Dose	Comments	References
1	*Withania somnifera* water-soluble extract	Normal Rat kidney Epithelial cells	1:1000 dilution of *Withania somnifera* capsule containing 2.5% of withanolide	*Withania somnifera* water-soluble extract prevented TNF-α-induced CCL5 increase, reduced CCL2 expression, and inhibited NF-κB activation; also effective against lipopolysaccharide-induced inflammation and aging-related disorders	[53]
2	*Withania somnifera* hydro-alcoholic extract	Male Sprague Dawley rats	500 mg/kg orally (for 60 days)	*Withania somnifera* hydro-alcoholic extract decreased glucose, inflammatory markers (IL-6, TNF-α, CRP), AMPK, malondialdehyde, and Bax (downregulation); upregulated Bcl-2, enhancing muscle health in aging.	[145]
3	*Withania somnifera* extract	Male albino rats	50, 100, and 150 mg/kg	*Withania somnifera* (150 mg/kg) enhances glutathione peroxidase, an antioxidant enzyme, showing potential in protecting against oxidative stress, especially in aging individuals, due to its antioxidant properties	[146]
4	*Withania somnifera* root extract	Wharton’s Jelly Mesenchymal Stem Cells	5, 10, 20, 40, and 50 mg/mL	*Withania somnifera* root extracts (5 mg/mL) upregulated proliferation marker Ki-67, while downregulating senescence marker p21, enhancing Wharton’s Jelly Mesenchymal Stem Cells growth	[147]
5	Withanolide A dimethyl sulfoxide Extract	*Caenorhabditis elegans*	100 µM	Withanolide A effectively reduced ROS levels, increased stress resistance, and extended lifespan in various *C. elegans* strains, while upregulating serotonin receptor and transporter genes, including the DA1814 strain	[148]
6	Withanone dimethyl sulfoxide Extract	Normal human fibroblasts (TIG-1, MRC5, and WI-38) cell line	5 µg/mL	Withanone protects normal cells, downregulates aging marker p21 WAF1, extends lifespan, reduces molecular damage, and upregulates proteasomal activity, suggesting antiaging potential	[149]
7	*Withania somnifera* Ethanol extract	Rotifers (*Phylum Rotifera*)	100 µM	*Withania somnifera* extract contained withaferin A, withanolide A, and withanolide B. Its effects on rotifers included changes in MCF, BSI, and toxicity	[150]
8	*Withania somnifera* Root Milk extract	T98G neuroglia cells	5.0 µg/mL	Adaptogens influenced 88 genes linked to stress responses, including neuronal and melatonin pathways, consistently upregulating key genes such as UCN, GNRH1, and RORA, suggesting stress protection and aging-related disorders	[151]
9	*Withania somnifera* Dried root extract	Human dermal fibroblasts	25, 50, and 100 µM	Withagenin A diglucoside reduces ROS, preserving collagen by inhibiting MMP-1 via MAPK, Akt, c-Jun, COX-2, and NF-κB pathways (downregulation). Withagenin A diglucoside also decreases IL-6 and IL-8 (downregulation), alleviating skin inflammation. These findings suggest Withagenin A diglucoside’s potential in cosmetics and pharmaceuticals for combating skin aging	[55]
10	*Withania somnifera* Dried leaf powder extract	Middle-aged female albino rats	200 mg/kg	Reduced anxiety and inflammation (downregulating Iba1, TNF-α, IL-1β, IL-6) in middle-aged rats, while upregulating astrocyte marker Glial fibrillary acidic protein	[72]
11	*Withania somnifera, Silybum marianum and Trigonella foenum-graecum* extract	RAW 264.7, C2C12 myoblasts and Primary human Osteoblast cells	10 µg/mL to 40 µg/mL	Dietary product of *Withania somnifera, Silybum marianum*, and *Trigonella foenum-graecum* extract downregulates TRAP and OC-related genes and inhibits osteoclast formation by downregulating RANK receptor pathways, including Src and p38 MAPK pathways, aiding osteoporosis treatment and bone mineralization	[152]
12	*Withania somnifera* Ethanol extract	C2C12 cells	100 µg/mL	*Withania somnifera* formulation upregulates MyHC-II, promotes protein synthesis via Akt pathway, and enhances myoblast differentiation through p38 MAPK/myogenin pathway, countering muscle atrophy markers	[61]
13	*Withania somnifera* leaf hydro-alcoholic extract	Male Wistar rats	10 mg/kg	*Withania somnifera* restores SIRT1 and NRF2 daily rhythms and phases, indicating potential circadian and antioxidant benefits in aging	[81]
14	*Withania somnifera* extract	*Drosophila melanogaster*	0.1% *w*/*w*	*Withania somnifera* extract improved survival, motor function, and neuronal health, and reduced damaged mitochondria, showing potential therapeutic benefits for Amyotrophic Lateral Sclerosis-like conditions	[153]
15	Withanolides A dimethyl sulfoxide Extract	*Caenorhabditis elegans*	2, 5, 25, and 50 µM	Withanolides A extended *C. elegans* lifespan (29%), lowered lipofuscin (aging marker), and upregulated neuroprotective factors (SGK-1, DAF-16, SKN-1, HSF-1), signifying antiaging and neuroprotective potential	[20]
16	*Withania somnifera* dried root ethanol extract	Tail tendons of male Wistar rats	100 mg	*Withania somnifera*, especially its ethanolic extract, reduced collagen glycation, AGE formation, and cross-linking, potentially through its antioxidant properties, offering therapeutic potential against diabetes and aging	[60]
17	Withanolides A dimethyl sulfoxide (DMSO) Extract	*Caenorhabditis elegans*	5, 50, 100, 250, and 500 μM	Withanolide A at 5 μM upregulated Serum and glucocorticoid regulated kinase-1 in the insulin/insulin-like growth factor-1 pathway, downregulating fat accumulation, and extended lifespan in *C. elegans* models, showing antiaging potential	[154]
18	*Withania* dimethyl sulfoxide (DMSO) Extract	Male Swiss albino strain mice	200 mg/kg	*Withania somnifera* upregulated genes such as KLK8 and factors such as MAP2c, enhancing dendritic growth, memory, and neuroprotection while reducing age-related neurodegeneration	[155]
19	*Withania somnifera* root extract	Charles Foster strain male rats	20 mg/kg and 50 mg/kg	The study supported *Withania somnifera* for mood stabilization, attributing anxiolytic and antidepressant effects to GABA-mimetic activity, aligning with clinical use in Ayurveda	[156]
20	*Withania somnifera* rootMethanol extract	*Drosophila melanogaster* males	0.1, 1 and 10% *w*/*w*	The study employed LRRK2^WD40^ mutant fruit flies as a marker for Parkinson’s disease, showing motor impairments and mitochondrial dysfunction. *Withania somnifera* root upregulated protective factors but also downregulated toxic ones	[157]
21	Withaferin A methanol extract	SH-SY5Y cells	50 nM to 1 µM	The study identified markers (NF-κB, inflammasome), genes (HDAC2), and factors involved in Alzheimer’s disease. NF-κB and inflammasome genes were upregulated, while HDAC2 was downregulated	[90]
22	*Withania somnifera* root extract	*Caenorhabditis elegans*	100 ng/ml	*Withania somnifera* root extract extended *C. elegans* lifespan, particularly in acr-16 mutants	[158]
23	*Withania somnifera* root extract powder (KSM-66)	HeLa cell line	10–50 µg/mL	*Withania somnifera* root extract enhances telomerase in HeLa cells, indicating potential antiaging effects	[159]
24	*Withania somnifera* root extract (KSM-66)	Dogs	15 mg/kg	*Withania somnifera* root extract might alleviate stress, anxiety, pain and enhance well-being in dogs	[160]

**Table 2:** Clinical trials of *Withania somnifera* in inflammation and aging (adapted from www.clinicaltrials.gov (accessed on 26 December 2023) [165], ANZCTR www.anzctr.org.au/ (accessed on 26 December 2023) [166], and ICTRP www.who.int/clinical-trials-registry-platform (accessed on 26 December 2023) [167].

Registry ID Number	Status	Phase	Number of Participants	Conditions or Disease	Objective	Dose	Reference
NCT05430685	Completed	Phase 2	60	Craving, stress, sleep, well being	Effect of *Withania somnifera* in sleep, stress, and food cravings of healthy college students	350 mg twice daily for 30 days	[168]
NCT04092647	Recruiting	Phase 2	80	Chemo Fog	Role of *Withania somnifera* in cognitive dysfunction	350 mg for 9 weeks	[169]
(Kulkarni, 2018)	Completed	Phase 1 & 2	50	Chronic Periodontitis	Role of *Withania somnifera* in serum C reactive protein and salivary antioxidant in chronic generalized periodontitis	500 mg twice daily for 1 month	[170]
NCT03596307	Unknown	NA	12	Healthy	Role of *Withania somnifera* in endurance exercise performance	180 mg for 2 days and 16 days	[171]
NCT05031351	Recruiting	Phase 2	75	Amyotrophic lateral Sclerosis	Safety of *Withania somnifera* in participants with amyotrophic lateral sclerosis	1088 mg and 544 mg for 8 weeks	[172]
NCT01793935	Completed	NA	68	Schizophrenia, schizoaffective disorder	To determine whether *Withania somnifera* can reduce psychopathology scores and stress scores in schizophrenia	500 mg for first week followed by 1000 mg for 12 weeks	[173]
NCT00761761	Completed	Phase 3	60	Bipolar disorder	Effect of *Withania somnifera* in bipolar disorder	250 mg for first week followed by 500 mg for 8 weeks	[174]
NCT03437668	Active, not recruiting	Phase 2 & 3	66	Schizophrenia	Role of *Withania somnifera* in persistent symptoms of schizophrenia	500 mg for 12 weeks	[175]
NCT01311180	Completed	Phase 2	120	Generalized anxiety disorder	Role of *Withania somnifera* in generalized anxiety disorder	250 mg in morning for 7 days and 250 mg twice a day for 7 weeks	[176]
NCT05610735	Not yet recruiting	Phase 1 & 2	72	Recurrent ovarian cancer	To determine the tolerance of *Withania somnifera* with liposomal doxorubicin	Administration of liposomal doxorubicin 40 mg/m^2^ on day 1 of a 28-day cycle for 4 cycles; 2 g, 4 g, and 8 g of *Withania somnifera* for 2 years	[177]
NCT03112824	Completed	NA	35	Craving, obesity, sleep disturbance, stress reaction	Role of *Withania somnifera* in weight loss	300 mg of *Withania somnifera* twice a day for 12 weeks	[178]
NCT05994794	Active, not recruiting	NA	40	Elevated S-adenosylhomocysteine	Role of dietary supplement of normal homocysteine levels in healthy adults	Dietary supplement of *Withania somnifera*, creatine, and apha-GPC	[179]
NCT05210218	Completed	NA	40	Obesity	Dietary supplement of cinnamon and *Withania somnifera* in weight loss	Cinnamon and *Withania somnifera* (300 mg and 150 mg) for 4 weeks	[180]
NCT04733924	Completed	NA	77	Immune health	Investigate the efficacy and tolerability in improve immunity and reducing respiratory tract infection	125 mg and 250 mg for 84 days	[181]
NCT03780621	Completed	Phase 1	16	Cognitive impairment	Effect of adaptogenic extract on cognitive impairment	550 mg Andrographis and 10 mg of withanolides	[182]
NCT04598243	Unknown status	Early Phase 1	70	Fibromyalgia, chronic fatigue syndrome	Ribose, *Withania somnifera*, Rhodiola, Licorice, Schisandra, and Green Tea Extract for treating fibromyalgia and chronic fatigue syndrome	Herbal combination of Ribose, *Withania somnifera*, Rhodiola, liquorice, Schisandra, and Green Tea Extract	[183]
NCT04716647	Completed	NA	28	COVID-19	Investigate the feasibility of ayurveda in treating COVID-19	*Withania somnifera*: 250 mg to 5 g; Giloy: 500 mg to 1 g; Tulsi: 500 mg^−1^g	[184]
NCT05602389	Completed	NA	186	Stress	Investigate the role of multi-herb formulae and *Withania somnifera* root formula in stress	700 mg daily for 60 days	[185]
NCT01125501	Withdrawn	NA	0	Metabolic Syndrome	Evaluate the effects of Protandim on protein profile changes and markers of inflammation and oxidation in subjects (40–60 years of age) with metabolic syndrome	One capsule containing protandim (derived from the botanical extracts *Bacopa monniera*, *Silybum marianum*, *Withania somnifera*, *Camellia sinensis*, and *Curcuma longa*) daily for 30 days	[186]
NCT03262805	Completed	NA	73	Knee Osteoarthritis	Evaluate the efficacy of Lanconone^®^ (E-OA-07) in physical activity-related pain—LEAP study	1000 mg twice daily of Lanconone (Shyonak, *Withania somnifera*, Shunthi, Guggul, Chopchini, Rasna, and Shallaki) for 4 weeks	[187]
NCT02027467	Completed	NA	23	Healthy	Investigate the effect of StemAlive^®^ supplement on the levels of stem cells in human volunteers (hematoalive)	Three capsules of StemAlive^®^ (green tea, *Withania somnifera*) twice daily for 14 days	[188]
NCT02172625	Completed	NA	40	Oxidative Stress	To examine the impact of Protandim supplementation on oxidative damage and athletic performance	One pill daily for 90 days of pritandim (675 mg) containing Bacopa extract 150 mg; milk thistle 225 mg; *Withania somnifera* 150 mg; green tea 75 mg; turmeric 75 mg	[189]
NCT00719953	Completed	Phase 4	30	Elderly memory impairment	To assess the efficacy of Cognitex	One capsule thrice a day containing 600 mg GPC, 100 mg PS-omega 3, 20 mg vinpocetine, 50 mg uridine-5′-monophosphate (disodium), 550 mg plant extracts (150 mg wild blueberry, 125 mg *Withania somnifera*, 150 mg grape seed, 125 mg hops, ginger and rosemary)	[190]
NCT05339958	Completed	NA	112	Hair Thinning	To assess the effectiveness and safety of a novel dietary supplement containing botanical ingredients for hair thinning in men over the course of six months of continuous daily usage	Four capsules of Nutrafol^®^ Men (Sensoril *Withania somnifera*, BCM-95 BioCurcumin, USPlus Saw Palmetto, EVNolMax 20% Tocotrienol/Tocopherol complex, Bioperine (piperine), Cynatine HNS (solubilized keratin), and Capsimax (capsaicin)) once daily for 6 months	[191]
NCT06065241	Active, not recruiting	NA	40	Aging Inflammation	To investigate whether the botanical formulation, LLP-01, has a significant clinical effect on proteomic inflammatory biomarkers and epigenetic changes in healthy, older individuals	Two LLP-01 capsules containing 1000 mg (extracts from *Withania somnifera*, *Rosmarinus officinalis*, *Curcuma longa*, *Cotinus coggygria*, *Panax ginseng*, *Cordyceps militaris*, *Camellia sinensis*, *Cotinus coggygria*, and *Piper nigrum*) for 60 days	[192]
NCT02920125	Completed	Phase 3	96	Coronary artery disease, cerebrovascular disease, ischemic heart disease, deep vein thrombosis, peripheral arterial diseases, and vascular disease	To evaluate result of Ayurvedic SUVED & Reimmugen (Colostrum) treatment on vascular disease, CAD, CVA, and DVT	500 mg of SUVED capsule (*Terminalia Arjuna, Withania somnifera, Terminalia chebula, Cyperus rotundus, Apium graveolens, Vitis vinifera, Piper longum, Fagonia Arabica, Emblica officinalis, Terminalia belerica, Nymphaea stellate, Punica granatum, Bacopa Monnieri*) for 3 months	[193]
NCT05831241	Recruiting	Phase 4	45	Sexual health	To determine the effect of *Withania somnifera* extract (Capsule KSM-66) on sexual health in healthy women	300 mg twice daily of KSM-66 capsule for 8 weeks	[194]
NCT05840731	Recruiting	Phase 4	45	Sexual health	To investigate the role of *Withania somnifera* extract (Capsule KSM-66) in improving sexual health in healthy men	300 mg twice daily of KSM-66 capsule for 8 weeks	[195]
ACTRN12623000287639p	Not yet recruiting	Phase 2	100	Stress	To investigate effectiveness of herbal formulations for stress	Compare stress reduction: three tablets (Ziziphus, Passionflower, Lemon balm, Chamomile) vs. twp tablets (*Withania somnifera*, stigma) daily for 21 days	[196]
ACTRN12622001226796	Not yet recruiting	NA	100	Stress, anxiety	To assess the feasibility of herbal and nutritional medicines for managing post-flood stress and anxiety: a randomized controlled trial	Evaluate post-flood stress: Noble Kava (200 mg daily, two capsules twice), Executive B stress Formula (two tablets daily), *Withania somnifera* complex day (four tablets daily).	[197]
ACTRN12621001769875	Active, not recruiting	NA	40	Diminished ovarian reserve, infertility	To assess the effectiveness of adjunct naturopathy for pregnancy rates in women with diminished ovarian reserve compared to usual care alone: feasibility of a randomized controlled trial	Assessing naturopathic practice for women with diminished ovarian reserve: four consultations, five supplements (including *Withania somnifera*), a d personalized herbal medicine for 16 weeks.	[198]
ACTRN12621001551886	Completed	NA	120	High stress, fatigue, inflammation	To examine the efficacy and safety of a novel standardized *Withania somnifera* root extract in overweight middle-to-older age adults experiencing high stress and fatigue: a randomized, double-blind, placebo-controlled trial	*Withania somnifera* extract (one capsule taken orally, twice daily with or without food, delivering 400 mg a day for 12 weeks)	[199]
ACTRN12617000971336	Completed	NA	50	Energy and vitality, hormonal changes, wellbeing	To examine the impact of *Withania somnifera* supplementation on testosterone levels and vitality in healthy, overweight men	Two tablets daily (containing 10.5 mg of withanolide glycosides) for 8 weeks	[200]
ACTRN12617000698370	Completed	NA	78	Stress, cognitive performance, anxiety	To examine the effects of Ionix Supreme on stress, mood, energy, and anxiety	Ionix Supreme liquid Oral liquid, 60 mL daily for 4 weeks, ingredients include Chinese Wolfberry, *Withania somnifera*, vitamins and minerals	[201]
ACTRN12615000324516	Active, not recruiting	Phase 3/Phase 4	180	Menopause	To evaluate the safety and efficacy of two herbal formulations in reducing menopausal symptoms in otherwise healthy women.	Capsule Twice daily: Tinospora, Asparagus, *Withania somnifera*, Commiphora. Fenugreek capsule, 300mg extract, twice daily for 3 months	[202]
CTRI/2022/11/047340	Not Yet Recruiting	NA	60	Alcohol related disorders	To investigate *Withania somnifera* as a supplementary therapy for treatment of alcohol use disorder	*Withania somnifera* 250 mg two tablets before bedtime for 90 days	[203]
CTRI/2023/07/054711	Completed	Phase 3	60	Generalized anxiety disorder	To study the effects of *Withania somnifera* in patients with stress and anxiety having cardiovascular comorbidities	*Withania somnifera*: 300 mg twice a day after meals	[204]
CTRI/2023/07/054940	Open to Recruitment	Phase 3/ Phase 4	60	Iodine-deficiency hypothyroidism	To evaluate the safety and efficacy of *Withania somnifera* and selenium combination as a supplement in patients with subclinical hypothyroidism	*Withania somnifera* 500 mg and selenium 40 mcg: one capsule once a day after dinner for 90 days	[205]
CTRI/2022/11/047539	Completed	NA	68	Hair health	Safety and efficacy of *Withania somnifera* on hair health	One to two drops of *Withania somnifera* topical formulation daily for 75 days	[206]
-	Completed	NA	50	Quality of life and cardiorespiratory endurance	Efficacy of *Withania somnifera* in improving quality of life and cardiorespiratory endurance in human athletic adults	300 mg twice daily of KSM-66 capsule for 12 weeks	[207]
-	Completed	-	64	Stress and anxiety	To assess the safety and efficacy of a high concentration full-spectrum extract of *Withania somnifera* root in reducing stress and anxiety in adults	300 mg *Withania somnifera* or placebo capsule twice daily for 60 days	[208]
-	Completed	-	68	Male infertility	To evaluate *Withania somnifera* impact on sperm production in oligospermic males through a clinical pilot study	225 mg *Withania somnifera* capsule thrice daily for 12 weeks	[209]
-	Completed	-	50	Muscle strength	To examine the effect of *Withania somnifera* supplementation on muscle strength and recovery	300 mg twice daily for 8 weeks	[210]
CTRI/2015/07/006045	Completed	-	50	Female sexual dysfunction	To determine the efficacy and safety of a high concentration *Withania somnifera* root extract (HCARE) supplementation for improving sexual function in healthy females	300 mg twice daily for 8 weeks	[211]
	Completed	-	52	Chronic stress	To evaluate the safety and efficacy of a standardized root extract of *Withania somnifera* through a double-blind, randomized, placebo-controlled trial	300 mg twice daily for 8 weeks	[212]
CTRI/2019/11/021990	Completed	-	50	Mild cognitive impairment	To evaluate the efficacy and safety of *Withania somnifera* in improving memory and cognitive functioning in adults with mild cognitive impairment	300 mg twice daily for 8 weeks	[213]
CTRI/2016/05/006903	Completed	-	50	Subclinical hypothyroidism	To evaluate the efficacy and safety of *Withania somnifera* root extract in subclinical hypothyroid patients	300 mg twice daily for 8 weeks	[161]
-	Completed	-	60	Insomnia and anxiety	To determine the efficacy and safety of *Withania somnifera* root extract in patients with insomnia and anxiety	300 mg twice daily for 10 weeks	[214]
-	Completed	-	50	Mental alertness	To assess the safety, efficacy, and tolerability of *Withania somnifera* root extract on the improvement of general health and sleep in elderly people	300 mg twice daily for 12 weeks	[215]
CTRI/2016/04/006791	Completed	-	50	Cardiorespiratory endurance	To evaluate the efficacy and safety of *Withania somnifera* root extract in enhancing cardiorespiratory endurance in healthy athletic adults	300 mg twice daily for 8 weeks	[26]
CTRI/2019/10/021547	Completed	-	100	Perimenopause	To assess the efficacy and tolerability of an *Withania somnifera* root extract on the climacteric symptoms, quality of life, and hormonal parameters in perimenopausal women	300 mg twice daily for 8 weeks	[216]
CTRI/2016/05/006906	Completed	-	50	Overall well-being	To evaluate the effect of *Withania somnifera* root extract on improving sexual health in adult males	300 mg twice daily for 8 weeks	[195,217]
-	Completed	-	80	Poor sexual function	To evaluate the efficacy and safety of standardized *Withania somnifera* root extract in improving sexual function in healthy females	300 mg twice daily for 8 weeks	[218]
-	Completed	-	75	Stress, anxiety, and impeded sleep	To investigate the adaptogenic and anxiolytic effects of *Withania somnifera* root extract in healthy adults	250 mg/day and 600 mg/day twice daily for 8 weeks	[219]
